# Sig2BioPAX: Java tool for converting flat files to BioPAX Level 3 format

**DOI:** 10.1186/1751-0473-6-5

**Published:** 2011-03-21

**Authors:** Ryan L Webb, Avi Ma'ayan

**Affiliations:** 1 Department of Pharmacology and Systems Therapeutics, Systems Biology Center New York (SBCNY), Mount Sinai School of Medicine, New York NY 10029, USA

## Abstract

**Background:**

The World Wide Web plays a critical role in enabling molecular, cell, systems and computational biologists to exchange, search, visualize, integrate, and analyze experimental data. Such efforts can be further enhanced through the development of semantic web concepts. The semantic web idea is to enable machines to understand data through the development of protocol free data exchange formats such as Resource Description Framework (RDF) and the Web Ontology Language (OWL). These standards provide formal descriptors of objects, object properties and their relationships within a specific knowledge domain. However, the overhead of converting datasets typically stored in data tables such as Excel, text or PDF into RDF or OWL formats is not trivial for non-specialists and as such produces a barrier to seamless data exchange between researchers, databases and analysis tools. This problem is particularly of importance in the field of network systems biology where biochemical interactions between genes and their protein products are abstracted to networks.

**Results:**

For the purpose of converting biochemical interactions into the BioPAX format, which is the leading standard developed by the computational systems biology community, we developed an open-source command line tool that takes as input tabular data describing different types of molecular biochemical interactions. The tool converts such interactions into the BioPAX level 3 OWL format. We used the tool to convert several existing and new mammalian networks of protein interactions, signalling pathways, and transcriptional regulatory networks into BioPAX. Some of these networks were deposited into PathwayCommons, a repository for consolidating and organizing biochemical networks.

**Conclusions:**

The software tool Sig2BioPAX is a resource that enables experimental and computational systems biologists to contribute their identified networks and pathways of molecular interactions for integration and reuse with the rest of the research community.

## Background

BioPAX is a protocol for the specification and representation of cell signaling pathways, gene-regulatory networks, protein-protein interactions and other types of biomolecular interaction data [1]. There are several software tools that use the BioPAX format for pathway visualization and analysis for hypotheses generation. For example, the popular tool Cytoscape allows customizable visualization and easy navigation of different types of networks [2]. Cytoscape plug-ins, including the popular BiNGO [3], and other plugins such as BiNoM [4], and cPath [5] further extend Cytoscape's capabilities for pathway analysis, data visualization, and data integration. BiNGO is a plugin that statistically analyzes a set of genes and their corresponding Gene Ontology functional annotations to determine which functional categories are overrepresented in that gene set. BiNGO uses Cytoscape's visualization capabilities to display the results. BiNoM is a plugin that performs structural analysis of networks, identifying strongly connected components, paths and cycles. cPath is an interaction database that can be included in Cytoscape as a plugin. The cPath database is a central repository for pathway and interaction datasets from multiple sources including MINT [6], IntAct [7], Reactome [8], and BioGRID[9]. The plugin allows for data retrieval from the central cPath database via an XML Web Services API, using the Cytoscape visualization engine for viewing biochemical networks. Interaction data stored in cPath are in BioPAX format.

BioPAX is one of several specification protocols that have been developed in an attempt to formally characterize biochemical regulatory molecular interactions. Some of these other specifications include the Proteomics Standard Initiative Molecular Interactions format (PSI-MI) [10] and the Systems Biology Markup Language (SBML) [11]. There are tools for conversion of some of these data formats into BioPAX. The previously mentioned Cytoscape plugin BiNoM also allows for conversion between BioPAX, SBML, and CellDesigner formats. However, most biochemical interaction data is not stored in one of these formats already, but rather stored in flat files, Excel spreadsheets, as network diagrams, or as tables in PDF format. While there are commercial products available to help researchers transform their flat text files into a general OWL format, to date, there are no tools available to transform flat files into BioPAX format. Such a tool would be useful because there are many pathway databases and networks that need to be converted for data sharing and reuse. Additionally, biologists that identify new interactions or describe new pathways in publications and do not have the technical expertise to convert their interaction data into BioPAX format will be able to do so with the tool.

## Implementation

**Figure 1:**
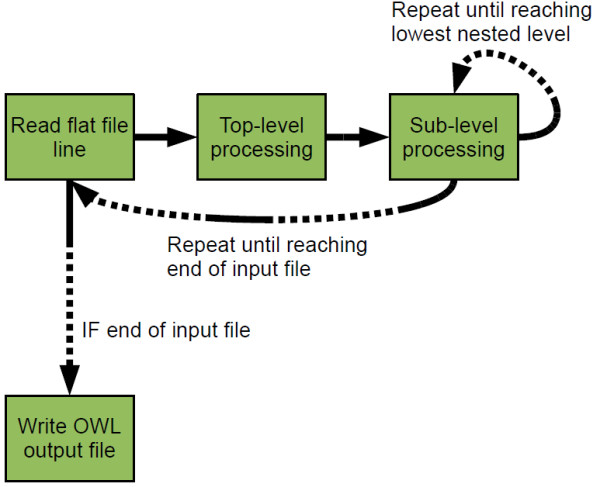
**A flow diagram of Sig2BioPAX**. The input file is processed by the Top-level processing unit and then by Sub-level units until reaching the lowest level of processing iteratively. Once all lines from the input file were processed the program outputs an OWL file.

We created a Java-based tool, Sig2BioPAX, for the purpose of converting cell signaling pathways and interaction network datasets into BioPAX. The software package uses the Jena Library[12], which includes data structures for representation and modification of RDF and OWL files. In the implementation, BioPAX classes are represented as Java classes. The classes contain variables which store the settings and details necessary for proper instantiation of those classes. Program control is passed through a tree of functions which adds new instances of classes as necessary to represent the data from the input file. Reading the input flat text file line-by-line, Sig2BioPAX adds nodes to the OWL model sequentially. Before adding a new node, the program traverses the model to make sure that no identical node (that is, a node having the same children) already exists. Finally, the newly created model is written to an OWL file using the Jena library functions. See Figure 1 for a flow-diagram of the internals of the Sig2BioPAX program.

There are a finite number of reaction types that can be processed and converted into the BioPAX format. These reactions are described in Table 1. Sig2BioPAX mainly supports cell signaling reaction types including protein-protein binding interactions, protein kinase phosphorylations and phosphatase dephosphoryations, guanine nucleotide exchange reactions, and ubiquitinations. Since BioPAX level 3 has added support for transcriptional regulatory interactions, Sig2BioPAX can be used to convert protein/DNA interactions in the form of transcription-factors binding to regulatory regions of genes. Sig2BioPAX categorizes the input reaction lines based on user-supplied rules. These rules determine the logic used to convert the flat files to the BioPAX level 3 format. The rules must be specified in a rules file using a custom language which is fully described in the program's instruction manual and also available within the GUI version of the program by clicking the question mark button. A default *rules.txt *is included with the program's release. Users may modify the included *rules.txt *or create their own rules file from scratch.

**Table 1:** A description of the reaction types processed by Sig2BioPAX

Reaction Type	Description
Binding	Molecules bind to form a complex
Kinase Phosphorylation	Kinase catalyzes addition of phosphate group to target molecule
Dephosphorylation	Catalyst initiates removal of phosphate group from target molecule
Guanine Nucleotide Exchange	GDP removed from complex and replaced with GTP
GTPase activating protein	GTP bound to compound becomes GDP
Ubiquitination	Ubiquitin molecule is added to target compound
Deubiquitination	Ubiquitin molecule is removed from target compound
Sumoylation	Small ubiquitin-related modifier is attached to target molecule
Cleavage with Phospholipase C	PLC cleaves PIP2 into IP3 and DAG
Cleavage on Cysteine	Deactivating cleavage on a cysteine residue of target protein
Inactivating Cleavage	Deactivating cleavage of target molecule
Activating Cleavage	Cleavage of pro-protein into active form
Protein-protein Interaction	Otherwise unspecified reaction between two proteins
Transcription	Protein activates or inhibits transcription of gene products
Transcription Factor Promoter Binding	Transcription factor and protein bind to create complex

One of Sig2BioPAX's accepted input parameter is the input file template type, which is a descriptor of the format of the input file. Four different input formats are accepted by the current version. The default type, *sig*, is described in Table 2. The second type, called *source_target*, forgoes describing the source and target types (i.e. kinase) and only describes the reaction type between the source and target (i.e. phosphorylation). The third template type is *tf_target*, used to describe transcription factor binding to the promoter of target genes as determined by ChIP-seq or ChIP-chip types of experiments [13]. The fourth template type is *sif*, or *simple interaction format*, which is a format specifying the target and source names and the interaction type. In the implementation, input templates are stored within the Line.java source file, which contains the function ReadLine(). This function is composed of IF statements, one for each input template type. In this code file, the testing condition for the IF statement are defined. The input line is parsed through a series of statements. By modifying the order and content of these statements, the user can customize the input templates.

**Table 2:** A description of an input line using the default input template, *sig*, and the meaning of the individual elements on the input line.

*sig *template: SN SH SM ST SL TN TH TM TT TL E TI ID	
SN	Source compound name
SH	Source Swiss-Prot human accession number
SM	Source Swiss-Prot mouse accession number
ST	Source Type of compound
SL	Source cellular location
TN	Target compound name
TH	Target Swiss-Prot human accession number
TM	Target Swiss-Prot mouse accession number
TT	Target Type of compound
TL	Target cellular location
E	Effect of source on target compound - Activating, inhibiting, or neutral
TI	Type of interaction
ID	Pubmed ID of reference article

Each element must be separated by whitespace, and the line is terminated by the newline character.

## Results

**Figure 2:**
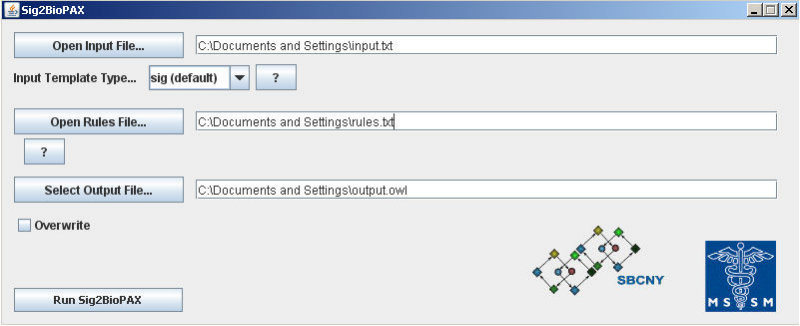
**A screenshot of the GUI version of Sig2BioPAX**. Users are provided with the ability to interactively specify the input file, the input file format, the rules file, and an output OWL file.

Sig2BioPAX is packaged as an executable JAR file. The tool is available in both command line and graphical user interface (GUI) versions. The command line version of the program, accessible by using the command line argument *-cmd*, accepts input parameters including the input file name, output file name, rules file name, option to overwrite output file, and an input-template type. In the command line version of the program, these options are passed as command line arguments. For example, to use input file foo.txt, output file bar.owl, the rules file rulesfile.txt, and the overwrite option, the command is: '**java -jar sig2biopaxv4.jar - cmd -in:foo.txt -out:bar.owl -r:rulesfile.txt -o**'. The *sig *input template type is the default input template type. In the GUI version of the program, the input, output, and rules files can be selected from a file browser and the other options can be set interactively (Figure 2). Sig2BioPAX can be downloaded from the Google Code project hosting site at http://code.google.com/p/sig2biopax/

We used Sig2BioPAX to convert several network datasets into the BioPAX format, and some of these networks are available on Pathway Commons [14], an international collaborative database of biomolecular pathways. The datasets we converted to BioPAX are original networks we extracted from the literature for the projects: the presynaptome [15], representing protein-protein interactions present in presynaptic nerve terminals of mammalian neurons, and the neuronal signalome [16], representing cell signaling interactions extracted from neuroscience literature describing combined cell signaling pathways in mammalian neurons, the adhesome [17], a network of interaction in focal adhesions; and a kinase-substrate network we constructed for the program KEA [18], kinase enrichment analysis, and ChEA [19], which stand for chip-seq/chip enrichment analysis.

## Conclusions

As the bulk and complexity of genome-wide molecular data increases, methods for sharing and exchanging data need to be further developed. Effective standard representation of data enables seamless data exchange across platforms, tools and databases. However, converting existing and new data into such exchange formats is not trivial. The Sig2BioPAX tool will further enable researchers to easily convert their flat file interaction data into the computable BioPAX format so that their data can be reused and interpreted by other researchers worldwide.

## Availability and Requirements

**Project name**: Sig2BioPAX

**Project home page**: http://code.google.com/p/sig2biopax/

**Operating system(s)**: Platform independent

**Programming language**: Java

**Other requirements**: Jena 2.6.2 or higher

**License**: GNU GPL

**Any restrictions to use by non-academics**: none

## Competing interests

The authors declare that they have no competing interests.

## Authors' contributions

AM initiated and managed the project. RLW implemented and tested the Sig2BioPAX Java tool. AM and RLW wrote the manuscript. All authors read and approved the final manuscript.
